# Montmorency Cherries Reduce the Oxidative Stress and Inflammatory Responses to Repeated Days High-Intensity Stochastic Cycling

**DOI:** 10.3390/nu6020829

**Published:** 2014-02-21

**Authors:** Phillip G. Bell, Ian H. Walshe, Gareth W. Davison, Emma Stevenson, Glyn Howatson

**Affiliations:** 1 Department of Sport, Exercise and Rehabilitation, Faculty of Health and Life Sciences, Northumbria University, Newcastle upon Tyne NE1 8ST, UK; E-Mails: phillip.g.bell@northumbria.ac.uk (P.G.B.); e.stevenson@northumbria.ac.uk (E.S.); 2 Health and Exercise Sciences Research Group, School of Sport, University of Stirling, Stirling FK9 4LA, UK; E-Mail: ian.walshe@stir.ac.uk; 3 Sport and Exercise Sciences Research Institute, University of Ulster, Northern Ireland BT52 1SA, UK; E-Mail: gw.davison@ulster.ac.uk; 4 Water Research Group, School of Environmental Sciences and Development, Northwest University, Potchefstroom 2520, South Africa

**Keywords:** recovery, strenuous exercise, muscle damage, *prunus cerasus*

## Abstract

This investigation examined the impact of Montmorency tart cherry concentrate (MC) on physiological indices of oxidative stress, inflammation and muscle damage across 3 days simulated road cycle racing. Trained cyclists (*n* = 16) were divided into equal groups and consumed 30 mL of MC or placebo (PLA), twice per day for seven consecutive days. A simulated, high-intensity, stochastic road cycling trial, lasting 109 min, was completed on days 5, 6 and 7. Oxidative stress and inflammation were measured from blood samples collected at baseline and immediately pre- and post-trial on days 5, 6 and 7. Analyses for lipid hydroperoxides (LOOH), interleukin-6 (IL-6), tumor necrosis factor-alpha (TNF-α), interleukin-8 (IL-8), interleukin-1-beta (IL-1-β), high-sensitivity C-reactive protein (hsCRP) and creatine kinase (CK) were conducted. LOOH (*p* < 0.01), IL-6 (*p* < 0.05) and hsCRP (*p* < 0.05) responses to trials were lower in the MC group versus PLA. No group or interaction effects were found for the other markers. The attenuated oxidative and inflammatory responses suggest MC may be efficacious in combating post-exercise oxidative and inflammatory cascades that can contribute to cellular disruption. Additionally, we demonstrate direct application for MC in repeated days cycling and conceivably other sporting scenario’s where back-to-back performances are required.

## 1. Introduction

Antioxidant supplementation has received growing attention for its role in aiding recovery from strenuous exercise [1,2,3] due to its ability to reduce cell damaging oxidative stress [4,5,6,7,8] and inflammation [9,10,11]. Specifically, the efficacy of antioxidant supplements such as ascorbic acid (vitamin C) [12,13,14], α-tocopherol (vitamin E) [15,16] and a range of functional foods with high antioxidant concentrations such as blueberries [17], beetroot [18,19] and cherries [10,20,21], have been investigated with regards to accelerating recovery or enhancing physical performance.

It has been suggested that foods with a high concentration of the flavanoids, anthocyanins, may be beneficial in recovery from exercise, which is proposed to increase the capacity for the inhibition of inflammatory cyclooxygenase (COX-1 and COX-2) enzymes and oxidative stress [6,22]. Anthocyanins are glycosides of their aglycon, cyanidin, and have been shown (*in vitro*) to be superior to α-tocopherol and the commercially available antioxidants butylated hydroxyanisole (BHA) and butylated hydroxytoluene (BHT) in the attenuation of lipid peroxidation [6]. Additionally, anthocyanins from Montmorency cherries have demonstrated similar anti-inflammatory properties to non-steroidal anti-inflammatory drugs (NSAID’s), such as ibuprofen and naproxen [22] and were superior to blueberries, cranberries and bilberries. Furthermore, a cell culture study in rat hepatocytes demonstrated anthocyanins inhibited reactive oxygen species (hydrogen peroxide) whilst up-regulating the expression of endogenous antioxidants glutathione reductase, glutathione peroxidase and glutathione *S*-transferase [23].

Montmorency cherries are a good candidate for recovery following intense exercise in humans because of their high concentrations of anthocyanins [5]. The first study to demonstrate accelerated exercise recovery used 9 days of Montmorency cherry supplementation (4 days pre-, on the day and 4 days post-exercise) [20]. In the 96 h following an eccentric-induced muscle damage protocol, supplementation with Montmorency cherries showed significant attenuation in the decline of isometric strength when compared with a placebo (4% *vs*. 22%). This was supported more recently by research demonstrating that 10 days of Montmorency cherry supplementation (7 days pre-, on the day and 2 days post-exercise) resulted in a more rapid recovery of isokinetic knee extensor force than an isoenergetic placebo following an eccentric-induced muscle damage protocol [21]. Despite these findings neither study was able to demonstrate changes in oxidative stress or inflammation, although a trend towards attenuated oxidative stress (protein carbonyls) was reported in the latter study with Montmorency cherries condition [21]. In addition to the aforementioned studies, attenuation of both oxidative stress and inflammatory responses to exercise with Montmorency cherries supplementation (8 days; 5 days pre-, on the day of and 2 days post-exercise) have been demonstrated following marathon running [10]. In the 48 h following marathon running, participants supplemented with Montmorency cherries showed significantly lower oxidative stress (thiobarbituric acid reactive substances [TBARS]) than the placebo counterparts and in addition, the inflammatory response (interleukin-6 [IL-6] and high-sensitivity C-reactive protein [hsCRP]) were significantly lower in the Montmorency cherries versus placebo group. The discrepancies in findings with regards to biomarkers of oxidative stress and inflammation between Howatson *et al*., (2010) [10] and Bowtell *et al*., (2011) [21] may be explained by the mode of exercise used in the studies. Whilst Bowtell *et al*., (2011) [21] used a protocol to induce stress via a mechanically challenging exercise (eccentric muscle actions), the marathon running used by Howatson *et al*., [10] induced stress via both mechanical and metabolic pathways. Exercise that involves maximal muscular contractions such as those performed in Bowtell *et al.*, (2011) [21] derives energy primarily from anaerobic pathways (ATP-PC system and anaerobic glycolysis) [24] and generally induces a high degree of mechanical stress on the muscle; whereas endurance exercise requires more prolonged energy turnover from aerobic sources (aerobic glycolysis and beta oxidation). Differences in energy pathway required impacts upon the magnitude and type of stress caused by the exercise with maximal muscular contractions causing greater mechanical damage (particularly when eccentric in nature) [25,26]. Running a marathon provides high mechanical stress through eccentric muscle actions and a high metabolic cost due to the requirements of sustained higher energy expenditure associated with this activity. This finding suggests that the positive effects of Montmorency cherries on recovery may be well suited to exercise with a high metabolic component.

To date, no study has investigated the effects of Montmorency cherry supplementation on recovery from exercise that induces stress almost exclusively through metabolic pathways. Cycle ergometry provides an exercise model with little or no eccentric component. Additionally, repeated days strenuous cycling (akin to road cycling stage races) provides a suitable exercise paradigm for investigating repeated days performance in a metabolically challenging exercise, that has the scope to cause temporary perturbations inflammation and oxidative stress. Based on the evidence from previous literature, it was hypothesised that Montmorency cherry supplementation would attenuate the oxidative and inflammatory responses to the metabolic stress imposed via cycling across 3 days. As a result, the aim of the study was to investigate the impact of Montmorency cherries on metabolically exercise-induced oxidative stress and inflammation across 3 days of simulated cycle road racing.

## 2. Experimental Section

### 2.1. Participants

Sixteen well-trained, male cyclists (mean ± SD age, height, mass, VO_2peak_ was 30 ± 8 years; 181.1 ± 6.7 cm, 76.5 ± 9.2 kg, 61.6 ± 10.4 mL·kg^−1^·min^−1^, respectively) were recruited to take part in the study following completion of a medical health-screening questionnaire. Volunteers were excluded from the study if they met any of the following exclusion criteria; current smoking habit, food allergy (discussed with research team), current use of any food supplements, history of gastrointestinal, renal or cardiovascular disease. Institutional ethical clearance was granted by the Northumbria University, Faculty of Health and Life Sciences Research Ethics Committee prior to any testing and written informed consent was collected from all participants following both verbal and written briefings on the requirements of the study.

### 2.2. Study Design

**Figure 1:**
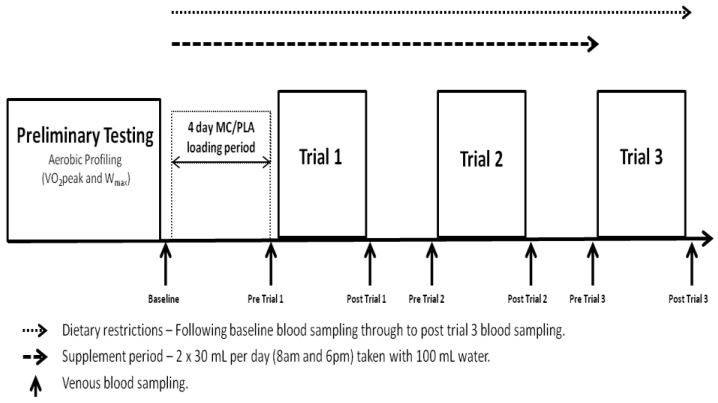
Schematic of testing protocol. Trials took place on consecutive days at 8:00 am following an overnight fast.

All elements of the study were conducted in laboratories accredited by the British Association of Sport and Exercise Sciences (BASES). The study utilised a double-blind, independent groups design and required participants to complete a trial period of 8 days, including 4 visits to the laboratory (Figure 1). Visit 1 consisted of a preliminary aerobic profiling (VO_2peak,_ W_max_) and familiarisation session, following which, the 16 participants were randomly, but equally divided into two groups (Montmorency cherry concentrate [MC] or Placebo [PLA]), that were counterbalanced by VO_2peak_ (63.1 *vs*. 60.2 mL·kg·min^−1^). Visits 2–4 were conducted on consecutive days following a 4-day supplement loading phase and required participants to complete the exercise protocol described. Each visit started at 7:45 am following a 10 h overnight fast. Blood samples for analysis of systemic markers of oxidative stress, inflammation and muscle damage were collected at baseline (prior to 4-day loading phase), pre-trial 1, post-trial 1, pre-trial 2, post-trial 2, pre-trial 3 and post-trial 3.

### 2.3. Supplementation and Dietary Control

Participants were provided with either MC or PLA supplementation after the initial visit and instructed to take the 30 mL of the supplement twice per day (8 am, 6 pm), for 7 consecutive days (4 days pre- and on each trial day [21]. All supplements were mixed with 100 mL of water prior to consumption. Independent analysis of MC [27] provided the following compositional data; Fat 0.028 mg·mL^−1^, Protein 31.47 mg·mL^−1^, Carbohydrate 669.4 mg·mL^−1^, Cholesterol < 0.01 mg·mL^−1^, Sodium 0.691 mg·mL^−1^, Calcium 0.137 mg·mL^−1^ and Iron 0.026 mg·mL^−1^. Additionally, according to the manufacturers guidelines (Cherry Active, Hanworth, UK), a 30 mL dose of MC is equivalent to approximately 90 whole Montmorency tart cherries and has been previously reported to contain 9.117 mg·mL^−1^ of anthocyanins [5,21,28]. The PLA supplement consisted of a commercially available, less than 5% fruit, cordial (Protein 20 mg·mL^−1^, Carbohydrate 260 mg·mL^−1^, Sodium 10 mg·mL^−1^, Fat–Trace, Fibre–Trace and Anthocyanins–Trace for colour), mixed with water and maltodextrin (MyProtein Ltd., Northwich, UK) until matched for carbohydrate content of the MC. An independent member of the department prepared and provided all supplements in opaque bottles to participants in order to maintain the double blind design.

Throughout the course of the trial period, participants were required to adhere to a low-polyphenolic diet requiring the avoidance of fruits, vegetables, tea, coffee, alcohol, chocolate, cereals, wholemeal bread and grains. Verbal and written explanations of foods to avoid and detailed examples of suitable foods and diets were provided. In order to assess for dietary compliance, participants completed food diaries for the duration of the study [28].

### 2.4. Exercise Protocol

Participants completed a simulated cycling road race at 8 am on visits 2–4. The cycling task was performed using a magnetically braked cycle ergometer (Velotron RacerMate, Seattle, WA, USA) and was a prolonged, high-intensity, stochastic performance previously used to simulate cycling road race demands [29]. Briefly, following a 10 min self-selected warm-up including 3 × 3 s sprints at 7, 8 and 9 min, participants completed 66 sprints lasting 5, 10 or 15 s, with a work (W) to rest ratio of 1:6, 1:3 or 1:1. Sprints were divided into 9 sets, with an active recovery period taking place between sets 1–2, 2–3, 4–5, 5–6, 7–8 and 8–9. All active recovery and rest periods were maintained at an intensity equal to 40%–50% W_max_. A further 9 min of sustained effort was incorporated into the task through the performance of 2 × 2 min and 1 × 5 min time-trials following the active recovery periods after sets 3, 6 and 9. During all time-trials, participants were encouraged to complete as much work as possible and the total duration of the trial was 109 min. Heart rate was continually recorded throughout each trial, using wireless heart rate telemetry (Polar Electro Ltd., Kempele, Finland). Additionally, power output (Velotron RacerMate, Seattle, WA, USA) data was collected at a frequency of 3 Hz and subsequently transformed into work performed (kJ). Throughout all trials, water was made available to participants *ad libitum* and strong verbal encouragement was provided. The exercise protocol is presented in Table 1.

### 2.5. Blood Sampling

Venous blood samples were collected from a branch of the basilic vein in the anti-cubital fossa region using the venepuncture method. A total ~21 mL of blood was collected into di-potassium ethylene diamine tetra-acetic acid (EDTA 10 mL), sodium heparin (6 mL) and serum separator (5 mL) tubes, respectively. Tubes were immediately centrifuged at 2400× *g*, 4 °C for 15 min, prior to the supernatant being removed and stored in aliquots at −80 °C.

**Table 1:** Simulated road cycling race protocol (Vaile *et al.*, 2008) [29]. Work—Maximal effort sprint. Rest—Power at 40%–50% W_max_. ACT (Active recovery)-Power at 40%–50% W_max_. TT (Time Trial)—Sustained maximal effort.

10 minute warm up (Self-selected pace)		
Set Number	Sprint Frequency/Duration	Work to Rest Ratio
Set 1	12 × 5 s	1:6 (Work:Rest)
Set 2	12 × 5 s	1:3 (Work:Rest)
Set 3	12 × 5 s	1:1 (Work:Rest)
4 min ACT-*2 min TT*-4 min ACT		
Set 4	6 × 10 s	1:6 (Work:Rest)
Set 5	6 × 10 s	1:3 (Work:Rest)
Set 6	6 × 10 s	1:1 (Work:Rest)
4 min ACT-*2 min TT*-4 min ACT		
Set 7	4 × 15 s	1:6 (Work:Rest)
Set 8	4 × 15 s	1:3 (Work:Rest)
Set 9	4 × 15 s	1:1 (Work:Rest)
5 min ACT-*5 min TT*-5 min ACT		

### 2.6. Oxidative Stress

To assess oxidative stress, samples were analysed for aqueous phase lipid hydroperoxides (LOOH) via the ferrous oxidation of xylenol orange method (FOX 1), using a modification of the methods by Wolff (1994) [30] and Nourooz-Zadeh *et al*., (1994) [31]. Absorbance was read spectrophotometrically (U-2001, Hitachi, England) at 560 nm against a linear standard curve (range 0–5 µmol·L^−1^). The inter-assay and intra-assay coefficients of variation were <4% and <2%, respectively.

### 2.7. Inflammatory Indices

Plasma tumour necrosis factor-alpha (TNF-α), interleukin-1-beta (IL-1-β), interleukin-6 (IL-6) and interleukin-8 (IL-8) were analysed using a multiplex electrochemiluminescence assay and plate reader (Sector Imager 2400, Meso Scale Discovery, Rockville, MD, USA). The intra- and inter-plate coefficients of variation were; 6.8% and 9.5% (IL-1-β), 4.9% and 8.8% (IL-6), 3.41% and 8.5% (IL-8), 4.64 and 11.5% (TNFα) respectively. Serum high sensitivity C-reactive protein (hsCRP) was analysed using an immunoturbidimetric assay (Roche Modular P, Roche Diagnostics Ltd., Burgess Hill, UK). Inter- and intra-assay coefficients of variation were 4.7% and 0.7%,respectively.

### 2.8. Muscle Damage

Serum creatine kinase (CK) was analysed using a kinetic UV test (Olympus Analyser, Olympus Diagnostica GmbH, Hamburg) in line with the recommendations of the International Federation of Clinical Chemistry (IFCC). Inter- and intra-assay coefficients of variation were both 1.5%.

### 2.9. Statistical Analysis

All data was analysed using statistical software (IBM SPSS 20 for Windows, New York, NY, USA) and are reported as mean ± standard deviation. Blood indices were anlaysed using a group (MC v PLA) by time-point (baseline, pre-trial 1, post-trial 1, pre-trial 2, post-trial 2, pre-trial 3, post-trial 3) repeated measures analysis of variance (ANOVA). The ANOVA analyses for heart rate and work done during the time-trial phases included four fewer time-point levels (Trial 1, Trial 2, Trial 3). Mauchley’s Test of Sphericity was conducted to test for homogeneity of data and where violations were present; Greenhouse-Geiser adjustments were made. LSD *post-hoc* analysis was used, where necessary, to identify significant interaction effects. A significance level of *p* < 0.05 was set prior to all analyses. Where appropriate, data were normalised using percentage to account for differences in baseline measures.

## 3. Results

**Figure 2:**
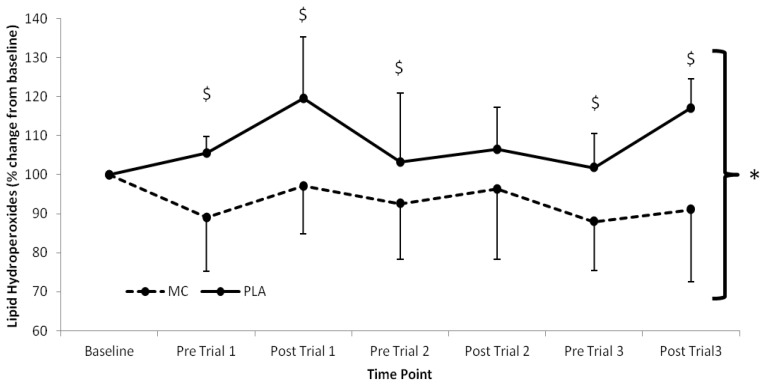
Serum lipid hydroperoxide responses (% Change from baseline) to Montmorency cherry concentrate (MC) and isoenergetic placebo (PLA). Absolute baseline values were 1.269 ± 0.085 and 1.189 ± 0.153 mmol mL^−1^ for MC and PLA respectively. * Significant group effect (*p* < 0.05), ^$^ Significant interaction effect (*p* < 0.05); values are mean ± SD.

Serum LOOH (Figure 2) showed significant time (*F*_(1,7)_ = 2.631, *p =* 0.022), group (*F*_(1,2)_ = 15.919, *p* < 0.001) and interaction effects (*F*_(1,7)_ = 3.956, *p* < 0.002). LOOH were elevated above baseline in the PLA group across the trial period, whilst the MC supplemented group demonstrated attenuated LOOH responses in the same period. The greatest difference between MC and PLA occurred following trial 3 where LOOH was 29.8% lower in the MC supplemented group.

**Figure 3:**
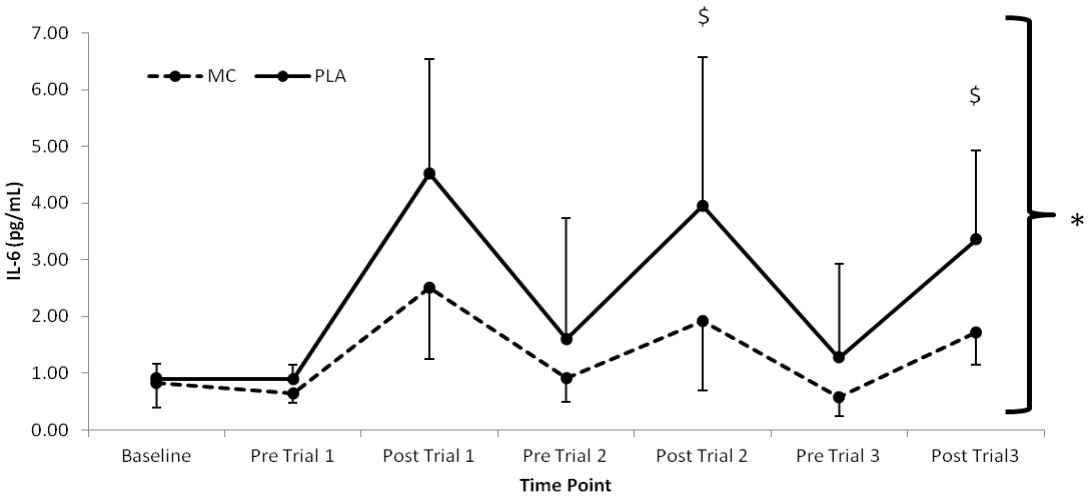
Plasma interleukin-6 responses (pg·mL^−1^) to Montmorency cherry concentrate (MC) and isoenergetic placebo (PLA). * Significant group effect (*p* < 0.05), ^$^ Significant interaction effects (*p* < 0.05); values are mean ± SD.

**Figure 4:**
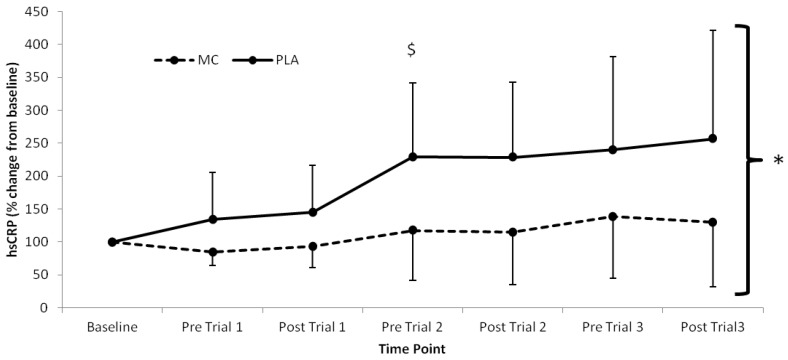
High-sensitivity CRP responses (% Change from baseline) to Montmorency cherry concentrate (MC) and isoenergetic placebo (PLA). Absolute baseline values were 0.889 ± 0.592 and 0.475 ± 0.191 pg·mL^−1^ for MC and PLA respectively. * Significant group effect (*p* < 0.05), ^$^ Significant interaction effects (*p* < 0.05); values are mean ± SD.

The pro-inflammatory marker, IL-6 (Figure 3), demonstrated time (*F*_(1,7)_ = 21.412, *p* < 0.001), group (*F*_(1,2)_ = 4.722, *p =* 0.047), and interaction effects (*F*_(1,7)_ = 2.803, *p* < 0.016) with lower values present in the MC *vs*. PLA group immediately following trial 2 (2.03 pg·mL^−1^) and trial 3 (1.63 pg·mL^−1^) (*F*_(1,7)_ = 2.803, *p* < 0.05). Significant time (*F*_(1,7)_ = 7.467, *p* < 0.001), group (*F*_(1,2)_ = 4.726, *p =* 0.047) and interaction (*F*_(1,7)_ = 2.534, *p* = 0.026) effects were also found for hsCRP, which was lower in the MC group across all time-points after baseline (Figure 4). A main effect of time was found for IL-8 (*F*_(1,7)_ = 15.411, *p* < 0.001), however there were no group or interaction differences present. No differences were found between IL-1-β or TNF-α (Table 2).

Muscle damage (CK) was not different between groups. However, CK did demonstrate a significant main effect for time (*F*_(1,7)_ = 3.213, *p* = 0.007), with a small mean increase of 77 U/I across the trial period (Table 2). Total work performed (kJ) during the time-trial phases decreased across each trial with a main effect for time (*F*_(1,3)_ = 7.667, *p* < 0.05) apparent. However, no differences between groups or interaction effects were found. Heart rate analysis during time-trial phases revealed no differences between groups or across the three trials (Table 3).

**Table 2:** Markers of inflammation and muscle damage not different between groups.

	Baseline		Pre-trial 1		Post-trial 1		Pre-trial 2		Post-trial 2		Pre-trial 3		Post-trial 3	
	Mean	SD	Mean	SD	Mean	SD	Mean	SD	Mean	SD	Mean	SD	Mean	SD
IL-1-β (pg mL^−1^)														
MC	0.09	0.15	0.10	0.17	0.06	0.08	0.05	0.09	0.14	0.20	0.10	0.15	0.08	0.12
PLA	0.27	0.47	0.11	0.14	0.20	0.22	0.20	0.29	0.19	0.29	0.16	0.20	0.13	0.18
IL-8 (pg mL^−1^) *														
MC	2.34	0.96	2.67	0.95	4.75	0.63	2.68	0.95	3.68	1.35	2.39	1.07	3.76	1.42
PLA	3.21	1.30	3.20	1.22	5.30	2.14	2.94	0.92	4.07	1.12	2.76	1.04	3.86	1.11
TNF-α (pg mL^−1^)														
MC	1.66	0.52	1.50	0.49	1.91	0.58	1.66	0.58	1.51	0.56	1.50	0.58	1.70	0.40
PLA	1.87	0.85	1.64	0.71	1.94	0.72	1.65	0.86	1.62	0.85	1.64	0.79	1.76	0.81
CK (IU/L) *														
MC	146.0	57.2	146.0	57.2	245.8	96.3	206.0	120.7	188.0	83.1	224.3	100.4	169.0	65.1
PLA	128.7	74.0	128.7	74.0	180.4	77.0	279.3	310.8	222.7	91.6	287.0	153.6	292.7	231.3

*: Significant main effect for time (*p* < 0.05); IL-1-β: Interleukin-1-beta; IL-8: Interleukin-8; TNF-α: Tumour Necrosis Factor-Alpha; CK: Creatine Kinase.

**Table 3:** Time trial data.

	Trial 1		Trial 2		Trial 3	
	Mean	SD	Mean	SD	Mean	SD
Heart Rate (b·min^−1^)						
MC	160.89	21.32	162.01	24.90	159.09	22.29
PLA	158.45	18.63	160.84	21.11	159.70	20.12
Work Performed (kJ) *						
MC	151.90	29.49	148.40	30.03	139.19	29.64
PLA	145.35	39.05	141.71	39.84	139.79	38.47

*: Significant main effect for time (*p* < 0.05).

Although food consumption during the trial phases was not subject to detailed dietary scrutiny, all food diaries were reviewed for compliance with dietary restrictions. Analysis of diaries demonstrated 100% adherence to dietary restrictions.

## 4. Discussion

This is the first study to investigate the impact of Montmorency cherries on systemic inflammatory and oxidative stress induced by a series of metabolically challenging cycling bouts. Despite both groups demonstrating a similar drop off in performance and no differences in time trial performance, the results show that both oxidative stress and inflammatory responses were attenuated with MC supplementation versus PLA.

Oxidative stress, as measured by LOOH, was lower in the MC and PLA groups across all time points following baseline measures. Interestingly, in the MC group, LOOH did not rise above baseline at any time point, although concentration did rise towards baseline following each bout. In contrast, LOOH in the PLA group remained above baseline values throughout the duration of the study. These findings concur with previous work; following marathon running [10], participants supplemented with MC showed no increases in TBARS (lipid peroxidation), whereas a PLA group demonstrated increased TBARS at 48 h post-exercise. The use of TBARS as a measure of lipid peroxidation has been criticised due to a lack of specificity in human models because the assay also reacts with prostaglandins, carbohydrates and both saturated and unsaturated non-functional aldehydes at 532 nm [32]. Conversely, LOOH have been shown to be a valid measure of post-exercise induced lipid peroxidation, and they are a reliable measure of cell membrane phospholipid damage [33]; consequently the data from the current study using LOOH suggest that lipid peroxidation is reduced with MC supplementation.

An interesting finding of note was the opposing changes in LOOH from baseline to pre-trial 1 between groups, whereby LOOH was reduced in the MC group by ~11% and raised in the PLA group by ~5%. This suggests that the antioxidant capacity of MC may have influenced redox balance and antioxidative capacity prior to exercise as other potential sources of dietary antioxidants were excluded from the diet. Although this is somewhat speculative, the removal of potential sources of antioxidants in the PLA group diet may have reduced antioxidant status and resulted in conditions suitable for increased lipid peroxidation. Antioxidant status was not measured in the current study, however, previous work using Montmorency cherries has demonstrated that an increase in oxidative stress can become apparent at the same time point that total antioxidant status falls below baseline levels [10], hence providing supporting for our supposition. The decreased LOOH in MC and increased LOOH in PLA across the duration of the study provide support that antioxidant capacity between groups was probably different.

Average increases of LOOH from pre-to-post trial in the MC group were lower than those in the PLA group (5% *vs*. 11%). The potential mechanisms for these observations are currently unknown, however the supplementation of MC make it possible that a number of events may have contributed to the findings; direct scavenging of free radical species responsible for damaging cells [34], formation of complexes resistant to free radical attack [35], up-regulation of endogenous enzymatic antioxidant defence systems [23] or lastly a synergistic combination of these [5,23,36].

Direct free radical scavenging alone would unlikely be responsible for the lowered LOOH found, as anthocyanins, the prominent antioxidant group within MC, are poorly absorbed [37,38] and are quickly excreted from the system [39,40] in humans. Additionally although an *in vitro* study has demonstrated the formation of complexes resistant to oxidative damage with anthocyanins mixing, this was only shown with DNA and not lipid structures, and is therefore not likely to have impacted on the LOOH levels in the current study [35]. The up-regulation of endogenous antioxidant enzyme defences seems to provide the most likely candidate mechanism of protection against lipid peroxidation. Anthocyanins have been shown to increase the expression of glutathione related antioxidant enzymes (glutathione peroxidase, glutathione reductase and glutathione-*S*-transferase) in rat hepatocytes [23]. It is possible that the consumption of MC triggered such a response and resulted in a greater capacity for the removal of free radical species responsible for the lipid peroxidation. Indeed, such enzymes have been implicated in both the prevention of lipid peroxidation (via reduction of hydrogen peroxide [H_2_O_2_]) and the removal of LOOH (via 2 electron reduction to inert alcohols and water) [33]. Although other mechanisms cannot be ruled out, this provides the most tenable explanation for the findings of reduced LOOH with MC consumption in the present study. In order to investigate this supposition, future research should look to address changes in both endogenous and exogenous antioxidant responses following MC supplementation.

The attenuation of post-exercise inflammation provides further support for the use of MC in the acceleration of recovery. In agreement with previous research, IL-6 and hsCRP [10] were both attenuated in the MC group. Interestingly, IL-6 increased following trial 2 and 3, which was greater in the PLA versus the MC group. Furthermore, there was little difference following trial 1, suggesting that MC has an inflammatory blunting response with cumulative bouts of metabolically strenuous exercise. Additionally, the attenuation of the rise in hsCRP, demonstrated a decreased secondary inflammatory response in the MC group. The reduction in both primary and secondary inflammatory responses to each trial in the MC group may be a downstream effect of reduced cell damage through oxidative stress during exercise. Conversely, the PLA group demonstrated a sharp increase in hsCRP following trial 1, which was maintained at an elevated level in accordance with increased LOOH across the remainder of the trial, a further indication that LOOH may have influenced the inflammatory state. It must also be noted however, that an increase in inflammation can contribute to a rise in oxidative stress [8], and as a result, it is conceivable that a reduction in inflammatory response was responsible for the observed attenuation in lipid peroxidation.

Both IL-6 and hsCRP values were considerably lower than those obtained following marathon running [10,41] and eccentric knee extensions [21], suggesting that the magnitude of the inflammatory response is significantly modulated by mechanically damaging components of exercise as opposed to the exclusively metabolic stress imposed in the current study. Nonetheless, the lower hsCRP response across the duration of the trial suggests that MC attenuates inflammation, which may have implications for clinical populations, particularly those who demonstrate low-grade chronic inflammation.

Results demonstrated drops in time-trial performances that were not different between groups across the three trials, providing evidence that the same degree of exercise stress was placed upon each group. Additionally, no differences in CK between groups were apparent, although a very small increase in CK was noted; suggesting a minimal amount of muscle damage was caused. As a result, greater confidence can be placed upon the proposed effects of MC on the oxidative stress and inflammatory responses discussed.

Oxidative stress and inflammation are linked with damage to the cell’s structure and function and the blunting of such responses following MC supplementation in the current study may be expected to influence functional performance. Although not a specific aim of this investigation, an acknowledged limitation is that no measure of functional performance, such as muscular strength or power, was included either prior to or following trials. In order to investigate this, future work should look to use a similar model of high-intensity cycling to identify the effect of metabolic stress on exercise-induced oxidative stress and inflammation, and the influence of these on specific measures of muscle function and performance.

## 5. Conclusions

This study is the first to demonstrate attenuated oxidative stress and inflammation responses following supplementation with MC in an exercise task that induced stress almost exclusively from metabolic pathways. We postulate that the observed reductions in oxidative stress and inflammation are associated with a greater ability to combat inflammation and the subsequent cascade of oxidative stress. Additionally, we demonstrate a direct application for MC during repeated days, high intensity cycling and conceivably other sporting scenarios where back-to-back performances cause appreciable levels of inflammation and oxidative stress. Further research should focus on a range of performance measures in order to identify the consequences of the exercise stimulus and the interaction of MC, oxidative stress, inflammation and human performance. Such research would provide application to other sporting scenario’s where there is a high metabolic stress component, such as team invasion sports.
